# A porous metal–organic framework (Pd-MOF) as an efficient and recyclable catalyst for the C–O cross-coupling reactions

**DOI:** 10.1038/s41598-025-97157-2

**Published:** 2025-04-16

**Authors:** Anjan Kumar, Ahmed M. Naglah, Vicky Jain, Suhas Ballal, Amit Sharma, Munthar Kadhim Abosaoda, Abhayveer Singh, T. Krithiga, Subhashree Ray, Ojas Prakashbhai Doshi

**Affiliations:** 1 Department of Electronics and Communication Engineering, GLA University, Mathura, 281406 India; 2 Department of Pharmaceutical Chemistry, College of Pharmacy, King Saud University, P.O. BOX 2457, 11451 Riyadh, Saudi Arabia; 3 Department of Chemistry, Faculty of Science, Marwadi University Research Center, Marwadi University, Rajkot, 360003 Gujarat India; 4 Department of Chemistry and Biochemistry, School of Sciences, JAIN (Deemed to be University), Bangalore, Karnataka India; 5 Department of Applied Sciences, Bharati Vidyapeeth’s College of Engineering, A4, Paschim Vihar, New Delhi, 110063 India; 6 College of Pharmacy, the Islamic University, Najaf, Iraq; 7 College of Pharmacy, the Islamic University of Al Diwaniyah, Al Diwaniyah, Iraq; 8 Centre for Research Impact & Outcome, Chitkara University Institute of Engineering and Technology, Chitkara University, Rajpura, 140401 Punjab India; 9 Department of Chemistry, Sathyabama Institute of Science and Technology, Chennai, Tamil Nadu India; 10 Department of Biochemistry IMS and SUM Hospital, Siksha ‘O’ Anusandhan (Deemed to be University), Bhubaneswar, 751003 Odisha India; 11 Arnold and Marie Schwartz College of Pharmacy and Health Sciences, Long Island University, Brooklyn, NY USA

**Keywords:** Pd-T-MOF, Coupling, Acid, MOF, Inorganic chemistry, Chemical synthesis, Chemistry, Catalysis, Catalyst synthesis, Homogeneous catalysis

## Abstract

This report outlines the development of a novel and efficient metal–organic framework (MOF) synthesized through a hydrothermal reaction using palladium acetate salt and trimesic acid as the organic ligand. A series of detailed analyses, including FT-IR, XRD, EDS, TEM, XPS, BET, ICP, and SEM, were performed to characterize the resulting MOF. These analyses confirmed the successful integration of Pd within the metal–organic framework structure. Nitrogen adsorption–desorption analysis assessed the porosity of the Pd-T-MOF metal–organic framework. The specific surface area was measured at 206.3 m^2^/g based on isotherms. Using the BJH method, the total pore volume was calculated as 0.4 cm^3^/g, with an average pore diameter of 2.8 nm. The catalyst demonstrated exceptional catalytic performance and stability in facilitating the C–O cross-coupling reaction. The proposed protocol offers several advantages, such as catalyst reusability, mild reaction conditions, high product yields ranging from 58 to 98%, and short reaction times between 30 and 120 min. Furthermore, the adaptable nanocatalyst (Pd-T-MOF) can be easily separated from the reaction mixture via centrifugation and reused across four successive cycles with only a slight decrease in efficiency.

## Introduction

Metal–organic frameworks (MOFs) are crystalline hybrid materials comprised of inorganic metallic clusters connected by organic linkers in a three-dimensional network^1–4^. These structures exhibit exceptional crystallinity, featuring nanometer-scale pores and cavities, which allow for significant internal volume and make them highly versatile for various industrial applications^5–7^. MOFs have gained attention for their ability to adsorb gases such as H₂, CH₄, and CO₂, as well as for their potential in catalysis a field of growing interest^8–10^. An important characteristic of MOFs is their incorporation of organic components within the solid framework ^11–13^. This feature not only contributes to their structural integrity but also provides opportunities to develop responsive, intelligent materials that can adapt to external stimuli^12^. Due to their high surface area and adjustable pore sizes, MOFs find extensive applications in the synthesis of catalysts, pharmaceuticals, and organic compounds^14^. However, challenges persist when it comes to heterogeneous metal-based catalysts, particularly regarding the leaching of costly or toxic metals into end products, which remains a concern in nanocatalysis^15^. Among various catalytic processes, transition metal-catalyzed carbon–carbon and carbon-heteroatom coupling reactions play a pivotal role in organic synthesis^16–18^. Palladium, in particular, is widely employed for such coupling reactions due to its high catalytic activity^19^. Homogeneous palladium catalysts like Pd(OAc)₂ and PdCl₂ are commonly used for these purposes^20^. However, their drawbacks such as challenges in separation and recovery, as well as their contribution to environmental pollutionlimit broader applications^18^. To overcome these limitations, the development of palladium nanocatalysts has emerged as an area of great interest, aligning with the principles of green chemistry^21^. In this context, using MOFs as catalytic platforms for organic reactions offers a promising avenue^22,23^. Their unique structure and functionality can address the shortcomings of traditional catalysts and pave the way for more efficient and sustainable catalytic processes. Metal–Organic Frameworks (MOFs) have garnered significant attention as efficient and eco-friendly catalysts in the chemical industry, thanks to their distinctive attributes, including high specific surface area, thermal stability, and exceptional structural and chemical versatility. Ongoing research in this area continues to unveil innovative applications for these versatile materials ^24–26^.

This research project focused on the synthesis of a Pd-based metal–organic framework (MOF) through the reaction of palladium (II) acetate and trimesic acid. The resulting crystalline phase, referred to as Pd-T-MOF, has emerged as a highly promising catalyst for promoting C–O cross-coupling reactions.

## Experimental

### Apparatus and materials

The starting reagents employed in this research for synthesizing Pd-T-MOF and conducting catalytic reactions included benzene tricarboxylic acid, aryl halide, phenol, palladium (II) acetate, KOH, MeOH, and a solvent. All reagents were of commercial-grade purity, procured from Aldrich and Merck, and used directly without undergoing further purification.

### Preparation of Pd-T-MOF

**Fig. 1:**
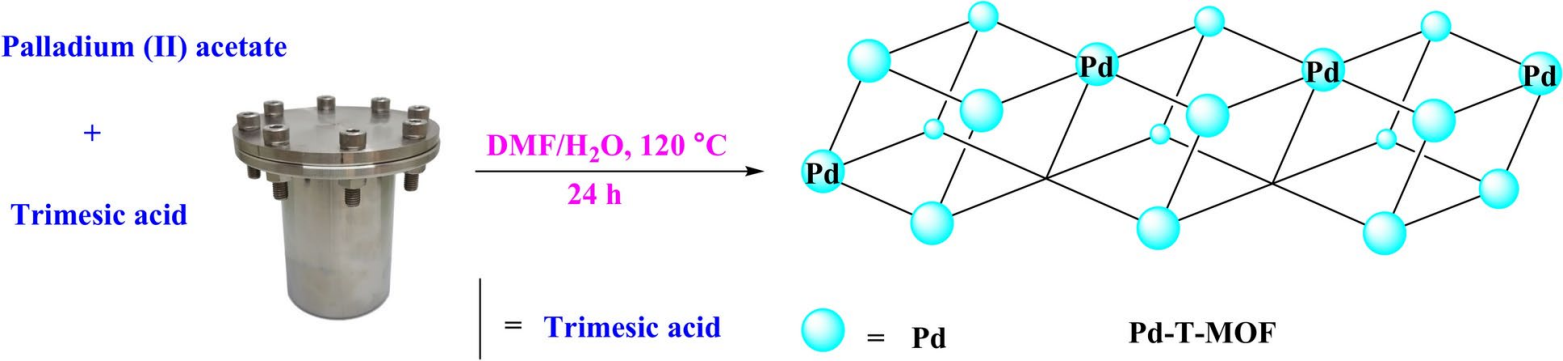
Synthesis of Pd-T-MOF.

In summary, 2 mmol of benzene tricarboxylic acid (T) was dissolved in a 12 ml mixture of dimethyl formamide (DMF) and ethanol in a 1:1 ratio. Separately, 4 mmol of palladium (II) acetate was dissolved in 12 ml of a DMF and water mixture with a 6:1 ratio. The two solutions were combined under stirring, and the resulting mixture was stirred at room temperature for 30 min. It was then transferred to an autoclave and heated at 120 °C for 24 h. The precipitate obtained was filtered, washed twice with 25 ml of DMF, and dried under vacuum at 60 °C for 5 h. The final Pd-T-MOF product (Light brown crystalline powder) was further dried under reduced pressure (Fig. 1).

### General procedure for C–O coupling reaction

**Fig. 2:**
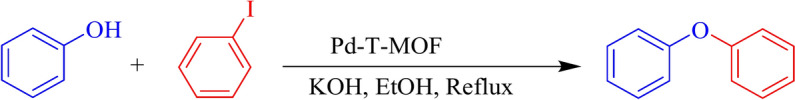
Synthesis of C–O bond catalyzed by Pd-T-MOF.

In the presence of 0.03 g of Pd-T-MOF, a combination of 1 mmol of aryl halide, 1 mmol of phenol, and 1.1 mmol of KOH was dissolved in MeOH and stirred under reflux conditions. The reaction progress was monitored using the TLC technique in hexane solvent. After the completion of the reaction, the Pd-T-MOF was separated by centrifugation, and the product was washed with ethyl acetate and H_2_O, while sodium sulfate was used as a water absorbent. Following the evaporation of the organic solvent, pure products were obtained (Fig. 2).

### Selected NMR data

**1-chloro-4-phenoxybenzene (**Table 2**, entry 11)**:^1^H NMR (400 MHz, DMSO): δ_H_ = 7.00- 7.29 (m, 10H) ppm.

**1-nitro-4-phenoxybenzene (**Table 2**, entry 5)**:^1^H NMR (400 MHz, DMSO): δ_H_ = 7.44 (s, 3H), 7.10 (m, 6H) ppm.

**1-methoxy-3-phenoxybenzene (**Table 2**, entry 8)**:^1^H NMR (400 MHz, DMSO): δ_H_ = 7.20 (d, 9H), 3.93 (s, 3H) ppm.

**1-bromo-4-phenoxybenzene (**Table 2**, entry 3)**:^1^H NMR (400 MHz, DMSO): δ_H_ = 7.0 (s, 4H), 6.7 (d, J = 12 Hz, 5H) ppm.

**Oxydibenzene (**Table 2**, entry 1)**:^1^H NMR (400 MHz, DMSO): δ_H_ = 8.3–8.5 (m, 5H), 7.9 (m, 5H) ppm.

**1-nitro-4-phenoxybenzene (**Table 2**, entry 9)**:^1^H NMR (400 MHz, DMSO): δ_H_ = 8.1 (m, 6H), 7.9 (m, 3H) ppm.

**Oxydibenzene (**Table 2**, entry 6)**:^1^H NMR (400 MHz, DMSO): δ_H_ = 8.0 (m, 5H), 7.4 (m, 5H) ppm.

**4-phenoxyphenol (**Table 2**, entry 4)**:^1^H NMR (400 MHz, DMSO): δ_H_ = 7.1–7.4 (m, 9H), 4.7 (s, 1H) ppm.

**1-methyl-4-phenoxybenzene (**Table 2**, entry 2)**:^1^H NMR (400 MHz, DMSO): δ_H_ = 7.0–7.2 (m, 9H), 2.1 (s, 3H) ppm.

**1-nitro-4-phenoxybenzene (**Table 2**, entry 13)**:^1^H NMR (400 MHz, DMSO): δ_H_ = 7.6 (m, 4H), 7.2 (s, 5H) ppm.

**1-methoxy-4-phenoxybenzene (**Table 2**, entry 7)**:^1^H NMR (400 MHz, DMSO): δ_H_ = 7.1 (m, 5H), 6.9 (m, 4H), 4.0 (s, 3H) ppm.

## Result and discussion

**Fig. 3:**
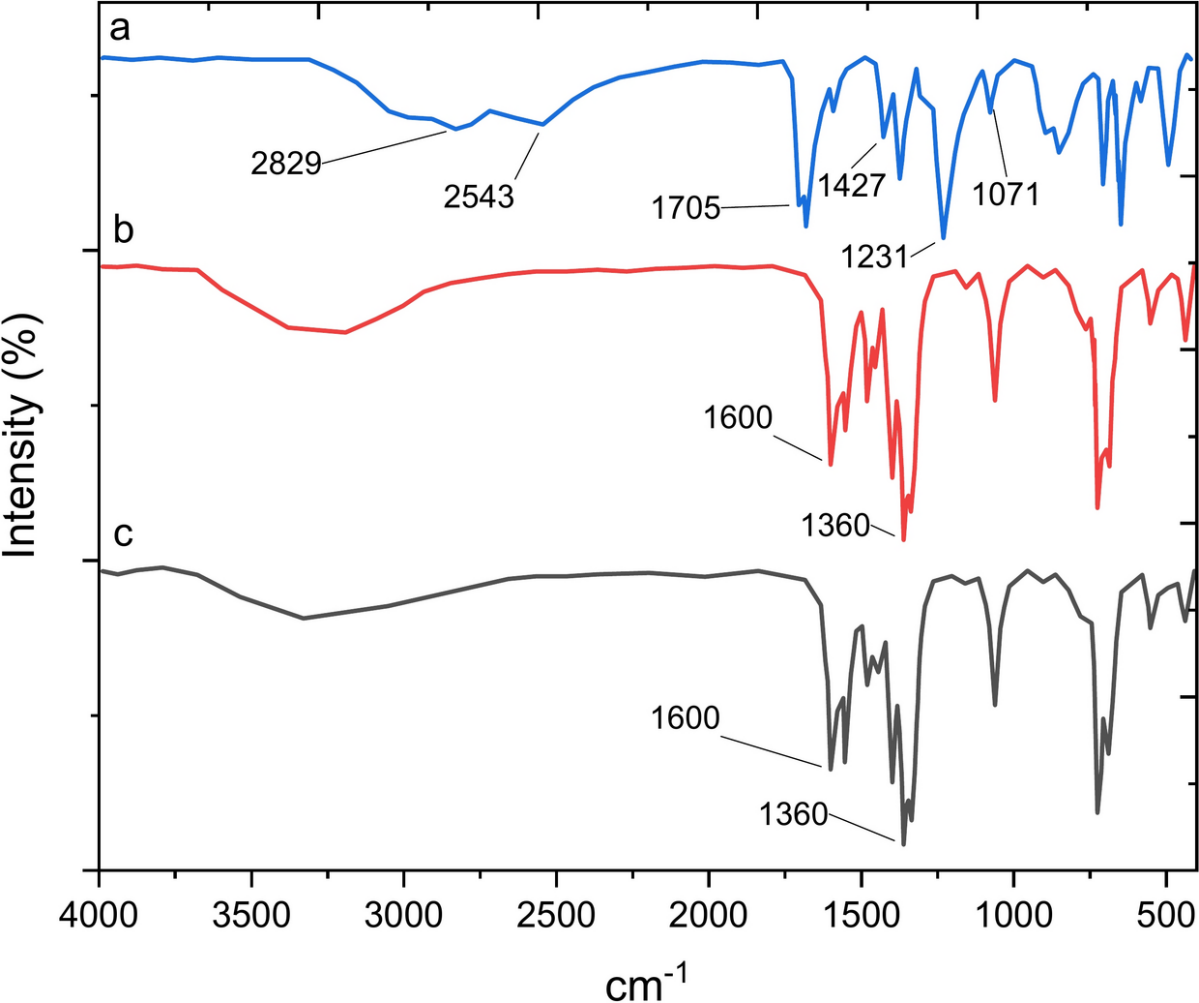
FT-IR spectrum of (**a**) benzene tricarboxylic acid, (**b**) Pd-T-MOF, (**c**) recovered catalyst.

The FT-IR spectra for pure benzene tricarboxylic acid, Pd-T-MOF, and the recovered catalyst are shown in Figs. 3a, 1b, and c, respectively. In the IR spectrum of benzene tricarboxylic acid (Fig. 3a), distinct peaks appear at 1705 cm^–1^, 1071 cm^–1^, and 1231 cm^–1^, corresponding to the stretching vibrations of C=O and C–O, as well as the bending vibration of O–H, confirming the presence of a carboxylic acid group. The symmetric and antisymmetric stretching vibrations of O–C =O groups are observed within the range of 1400–1500 cm^–1^. Moving to Fig. 3b, the characteristic absorption bands of benzene tricarboxylic acid within Pd-T-MOF shift to a lower wavenumber region (1600 cm^–1^) due to its coordination with palladium metal. Lastly, Fig. 3c indicates that the catalyst structure undergoes only minor alterations post-recovery, highlighting its stability within the reaction mixture.

**Fig. 4:**
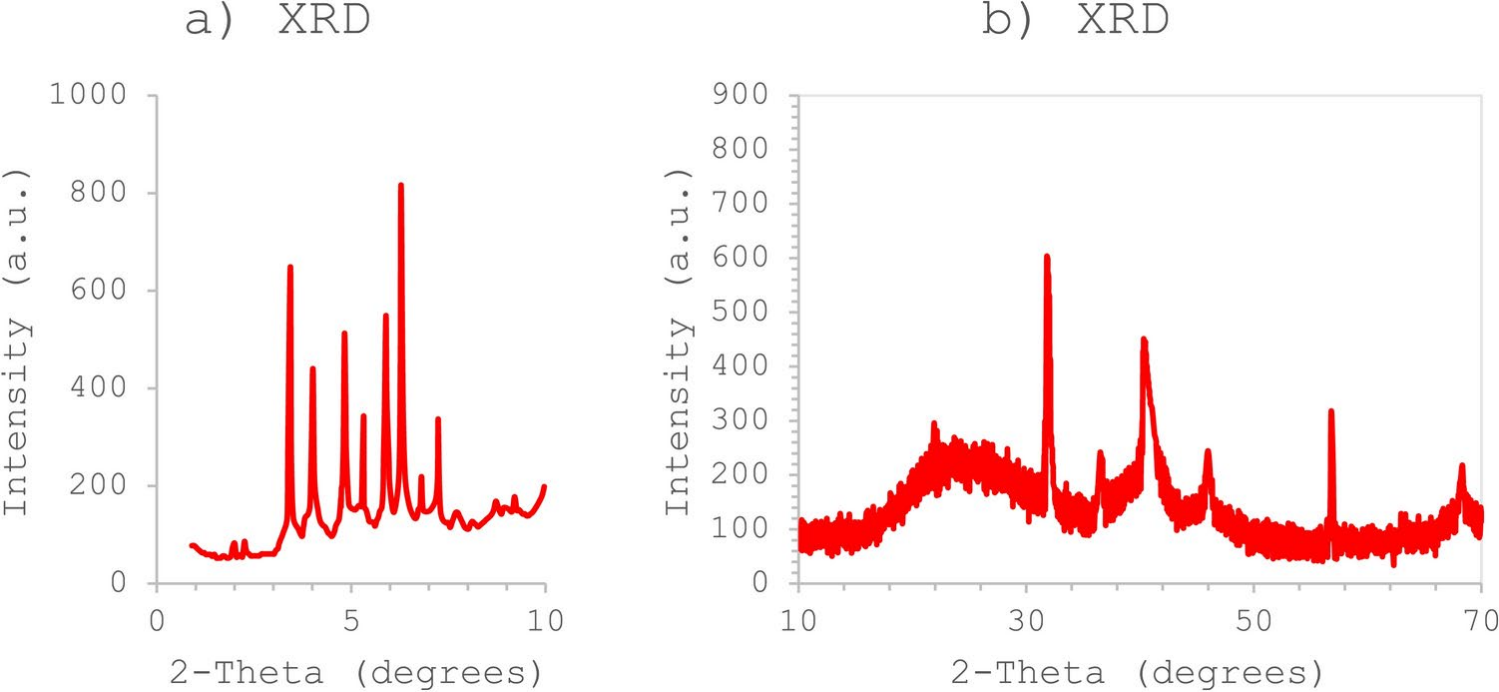
PXRD spectrum of Pd-T-MOF.

The low-angle powder X-ray diffraction (XRD) pattern of Pd-T-MOF is displayed in Fig. 4a. The patterns reveal multiple sharp peaks, likely attributed to the crystalline structure of the metal–organic framework. This confirms the successful nucleation of the porous Pd-T-MOF. The XRD patterns (Fig. 4b) were used to analyze the structural parameters of the grown pure Pd-T-MOF crystals. As shown in Fig. 4, the characteristic peaks for the crystallographic Pd-T-MOF can be found at relatively low diffraction angles (2) including 31.62°, 36.81°, 41.33°, 47.26° and 57.58°. From the XRD findings, it is evident that the Pd-T-MOF microspheres derived from Palladium (II) acetate exhibit distinct peaks, indicating the highly crystalline nature of the resulting nanoporous Pd-T-MOF microspheres.

**Fig. 5:**
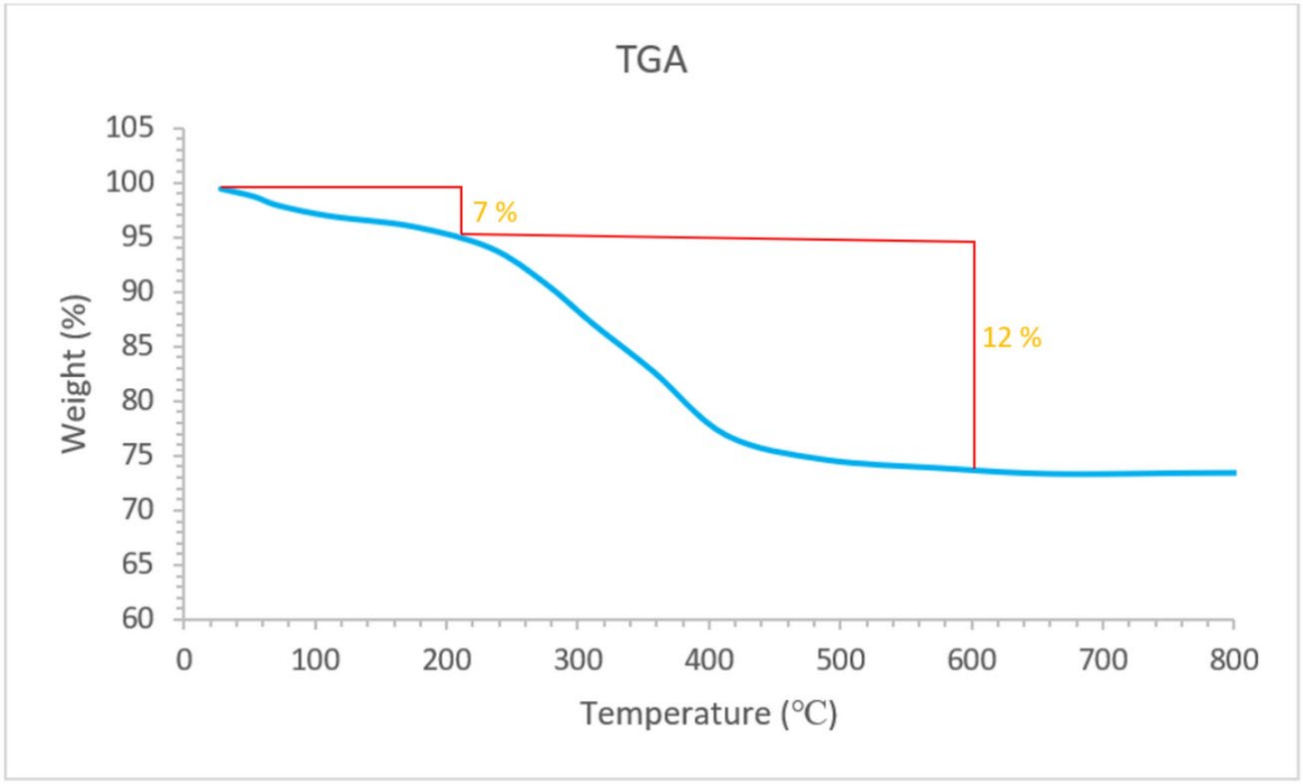
TGA diagram of Pd-T-MOF.

The TGA analysis of Pd-T-MOF is illustrated in Fig. 5. The curve indicates a 7% decrease in weight between 25 and 250 °C, attributable to the release of organic solvents and H_2_O. Additionally, the total mass decrease for Pd-T-MOF between 250–600 °C was approximately 12%, indicating the thermal stability of the synthesized Pd-T-MOF.

**Fig. 6:**
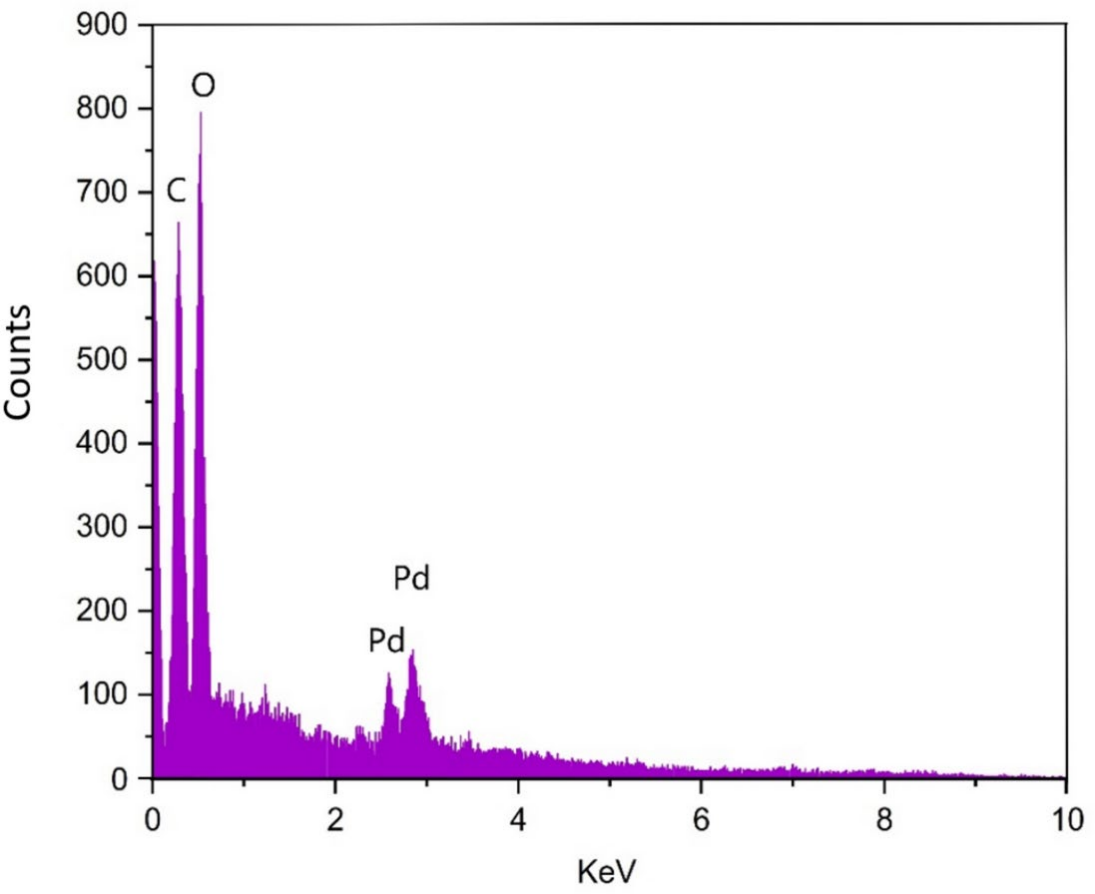
EDS spectrum of Pd-T-MOF.

Figure 6 demonstrates that the EDX analysis has validated the presence of oxygen, carbon, and palladium (Pd) elements in the synthesized material, confirming the successful production of Pd-T-MOF. The identification of Pd peaks in the spectrum confirmed the successful loading of Pd in Pd-T-MOF.

**Fig. 7:**
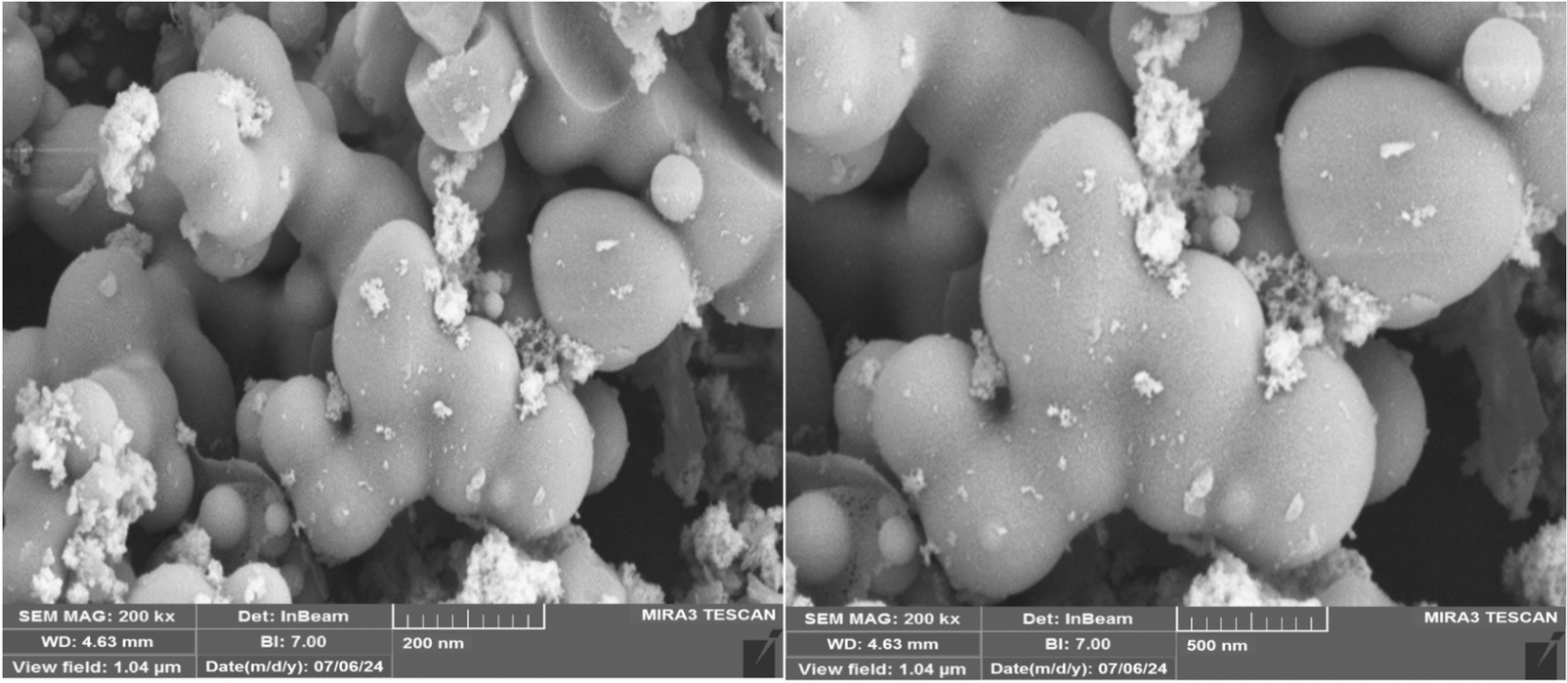
SEM spectra of Pd-T-MOF.

In Fig. 7, the SEM analysis reveals a consistent flat stick crystal structure in the Pd-T-MOF material. High-resolution imaging using SEM was employed to analyze the morphology of the Pd-T-MOF catalyst. The images obtained from this analysis, displayed in Fig. 7, indicate that the catalyst particles are predominantly less than 50 nm in size and exhibit mostly quasi-spherical shapes.

**Fig. 8:**
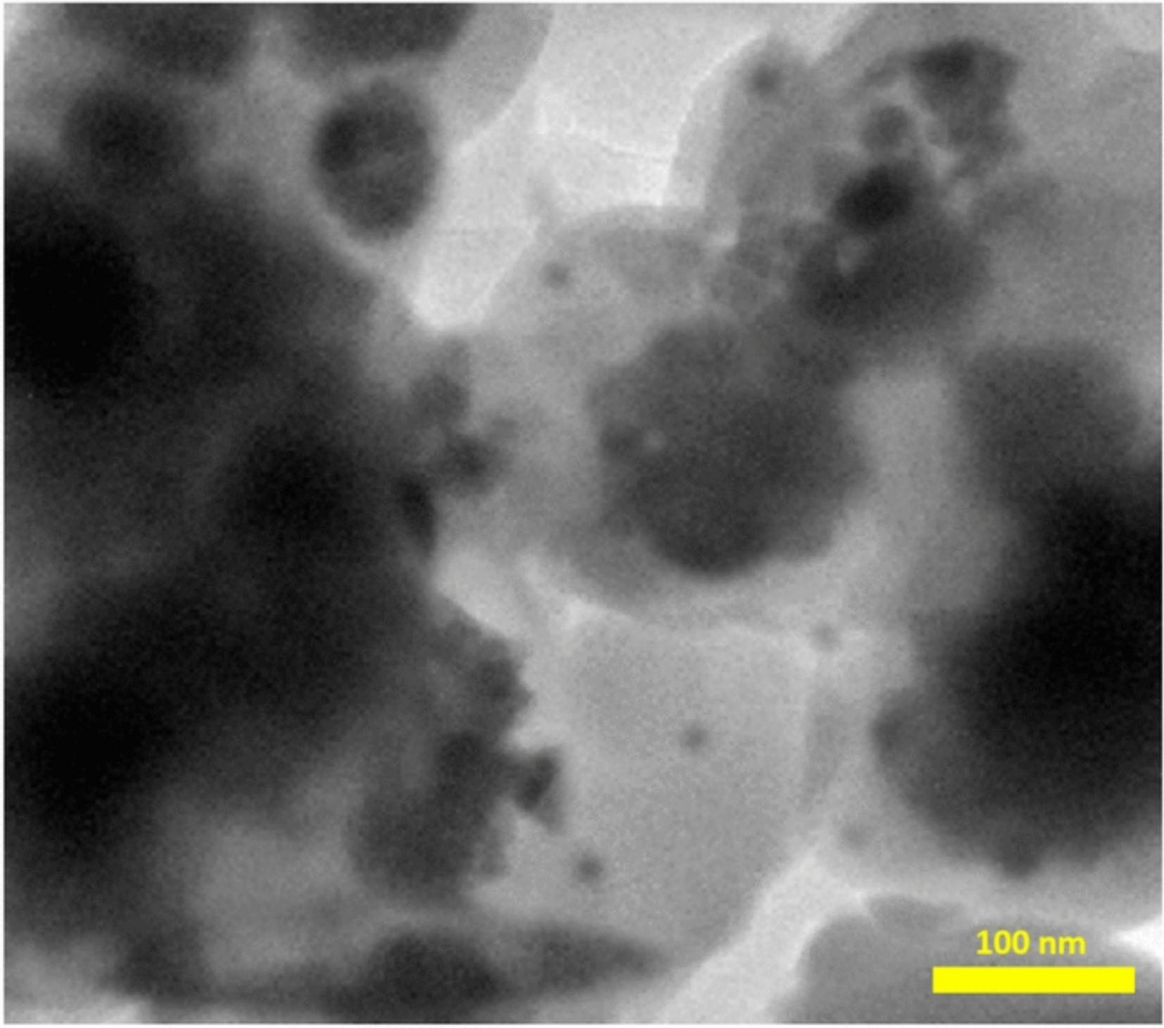
TEM spectra of Pd-T-MOF.

The morphology and structure of the synthesized metal–organic framework were examined using transmission electron microscopy (TEM). The findings were validated by the data derived from the TEM images presented in Fig. 8. As illustrated in Fig. 8, the synthesized metal–organic framework exhibits a spherical morphology with an average particle size of approximately 20 nm.

**Fig. 9:**
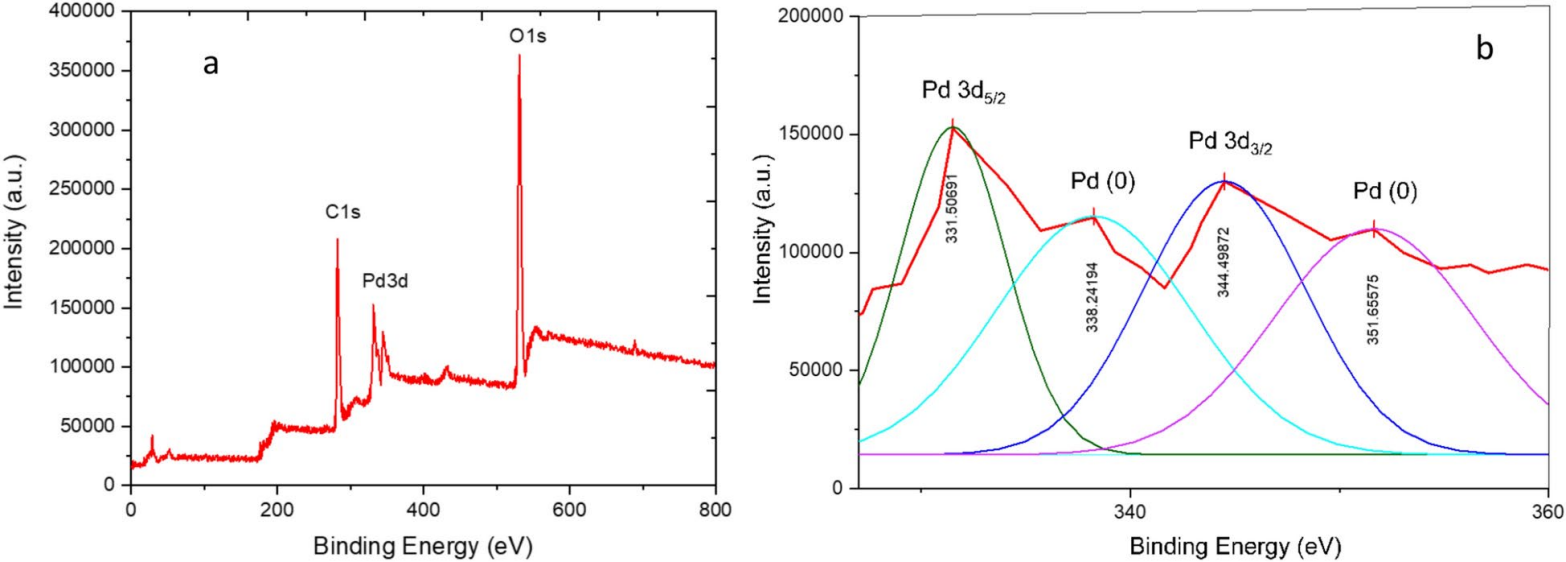
XPS spectrum of Pd-T-MOF.

Figure 9 illustrates the XPS spectrum of the synthesized Pd-T-MOF catalyst, highlighting peaks associated with oxygen, carbon, and palladium (also shown in Fig. 9a). The analysis of palladium via XPS is detailed in Fig. 8b. To identify its oxidation state, X-ray photoelectron spectroscopy (XPS) was employed, revealing two distinct binding energy peaks at 331.5 eV and 345.4 eV, corresponding to Pd 3d_5/2_ and Pd 3d_3/2_, respectively. This analysis confirms the structural composition of the synthesized Pd-T-MOF catalyst.

The study of palladium leaching from Pd-T-MOF involved ICP analysis, poisoning tests, and hot filtration tests. According to the results of ICP-OES analysis, the amount of palladium in fresh and reused Pd-T-MOF was found to be 0.21 × 10^–3^ mol g^–1^ and 0.19 × 10^–3^ mol g^–1^, respectively, indicating a very low level of metal leaching for this catalyst.

**Fig. 10:**
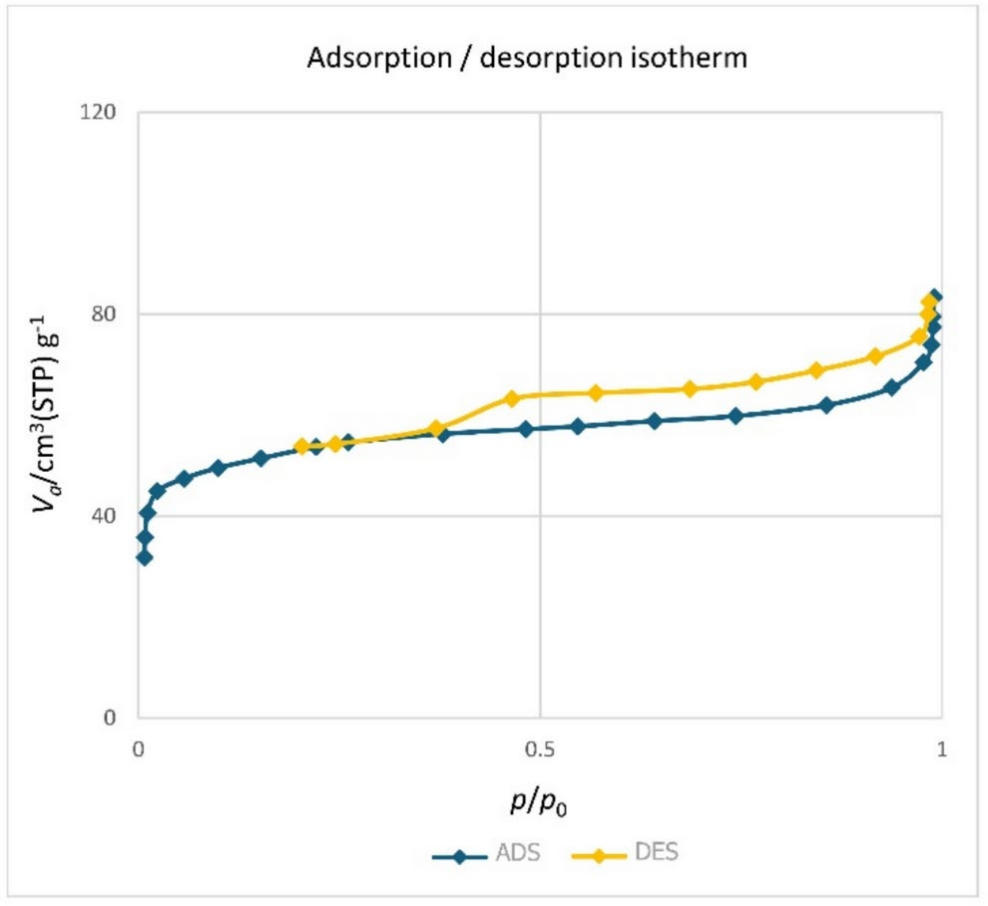
N_2_-adsorption isotherms of Pd-T-MOF.

The N_2_ adsorption/desorption analysis was employed to examine the porosity of the Pd-T-MOF metal–organic framework, as illustrated in Fig. 10. The results indicate that Pd-T-MOF exhibits a mesoporous structure. Using the BET method, the specific surface area of Pd-T-MOF was calculated to be 206.3 m^2^ g^–1^ based on N_2_ adsorption–desorption isotherms. Furthermore, the BJH method determined a total pore volume of 0.4 cm^3^ g^–1^ and an average pore diameter of 2.8 nm. These findings confirm that Pd-T-MOF qualifies as a porous material.

### Catalytic studies

The Pd-T-MOF was also studied for its catalytic activity in synthesizing di-aryl ethers via the carbon–oxygen coupling reaction of phenol with aryl halides (Fig. 2). Experiments were conducted to establish the optimal conditions for ether production by coupling phenol with iodobenzene as the model reaction (refer to Table 1) under different experimental parameters. Different amounts of the nanocatalyst were initially tested. Table 1, entry 1, shows that the desired reaction did not occur without the nanocatalyst, even after 6 h. With an increase in catalyst quantity, both the reaction rate and product yields showed improvement. After testing, it was determined that the optimal amount of Pd-T-MOF catalyst was 30 mg. Next, the model reaction was evaluated using various solvents while maintaining a constant catalyst quantity. Table 4 demonstrates that satisfactory outcomes were achieved in ethanol solvents. Ultimately, EtOH was chosen as the most appropriate solvent. Following this, the influence of different inorganic and organic bases on the model reaction in EtOH was explored, with the catalyst present at 30 mg. Alkali hydroxides proved to be more efficient as a base compared to other options, leading to enhanced reaction times and TOF values. Consequently, KOH was selected as the optimal base for the procedure. The reaction was then explored at various temperatures, with the most favorable outcome achieved under reflux conditions. Based on the studies mentioned above, the best conditions for synthesizing ethers were found to involve using ethanol as the solvent at reflux conditions, along with 30 mg of Pd-T-MOF and KOH as the base.

**Table 1:** Examining the ideal conditions for producing diphenyl ether in the presence of Pd-T-MOF.

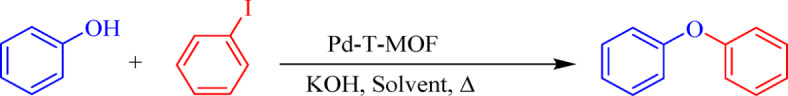						
Entry	Catalyst (mg)	Solvent	Base	Temperature (°C)	Time (min)	Yield (%)^a^
1	–	EtOH	KOH	Reflux	1 day	N. R
2	5	EtOH	KOH	Reflux	30	40
3	10	EtOH	KOH	Reflux	30	69
4	20	EtOH	KOH	Reflux	30	91
5	30	EtOH	KOH	Reflux	30	98
6	35	EtOH	KOH	Reflux	30	98
7	30	H_2_O	KOH	Reflux	30	44
8	30	EtOAc	KOH	Reflux	30	70
9	30	DMF	KOH	Reflux	30	81
10	30	Solvent-free	KOH	Reflux	30	89
11	30	EtOH	Na_2_CO_3_	Reflux	30	82
12	30	EtOH	Cs_2_CO_3_	Reflux	30	80
13	30	EtOH	K_2_CO_3_	Reflux	30	51
14	30	EtOH	KOH	60	30	76
15	30	EtOH	KOH	50	30	43
16	30	EtOH	KOH	25	30	N. R

^a^Isolated yield.

The methods employed for linking phenol with iodobenzene were also applied to facilitate the linking of other aryl halides, such as aryl bromides, chlorides, and iodides (refer to Table 2). The resulting products were achieved with high TOF values and outstanding yields within a short period. When Pd-T-MOF is present, the reactivity of aryl iodide in the carbon-oxcygen coupling reaction surpasses that of aryl bromide or chloride. In Table 2, the coupling of phenol with iodobenzene was completed in just 30 min, compared to 60 min for bromobenzenes and 45 min for chlorobenzene. Consequently, aryl iodides exhibit a higher reactivity than aryl chlorides and aryl bromides in the synthesis of ethers when Pd-T-MOF is present. This catalyst can selectively form bonds with aryl bromides or iodides instead of chlorides.

**Table 2:**
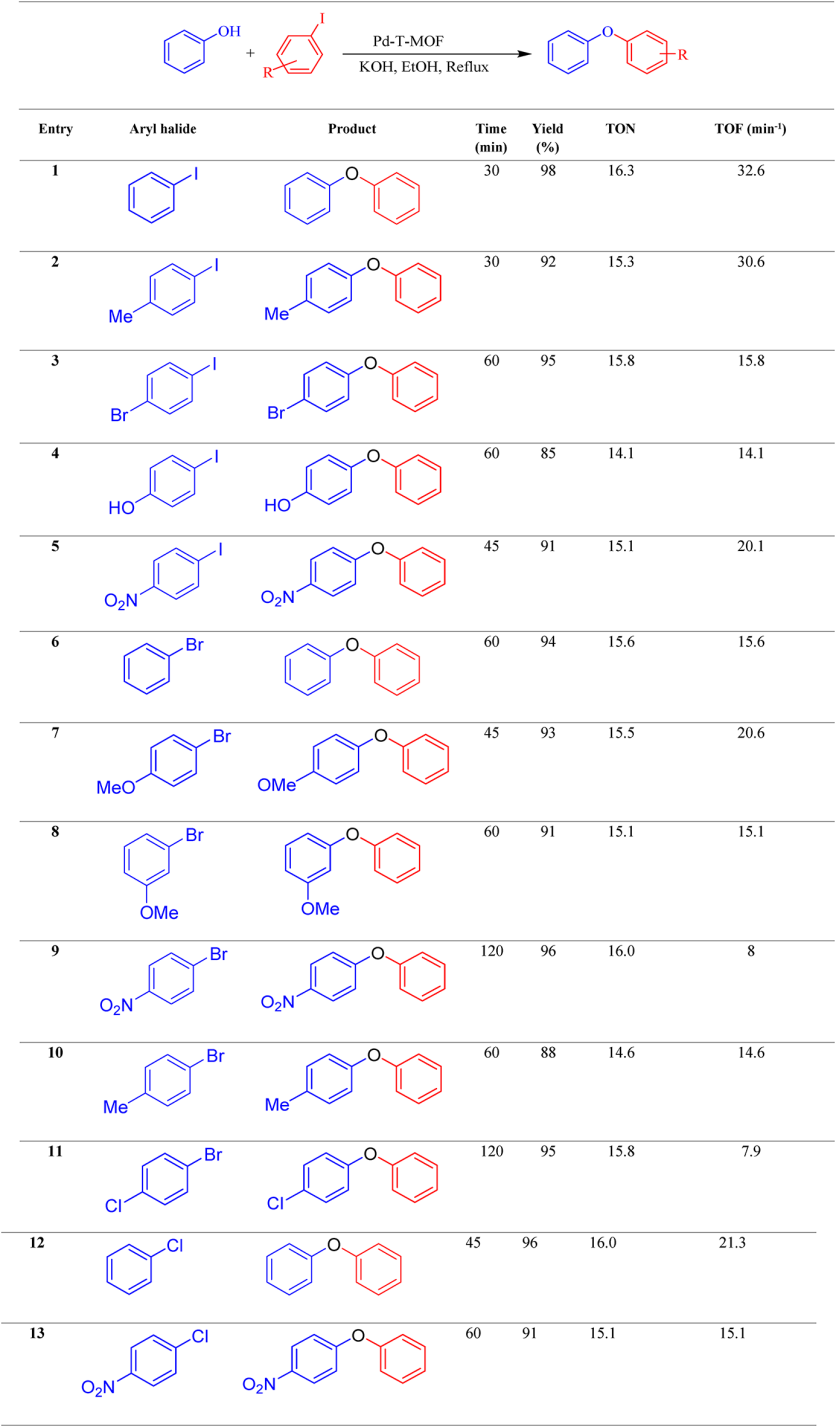
The Pd-T-MOF catalyst is used for catalyzing the C–O coupling reaction to synthesize di-aryl ethers.

**Fig. 11:**
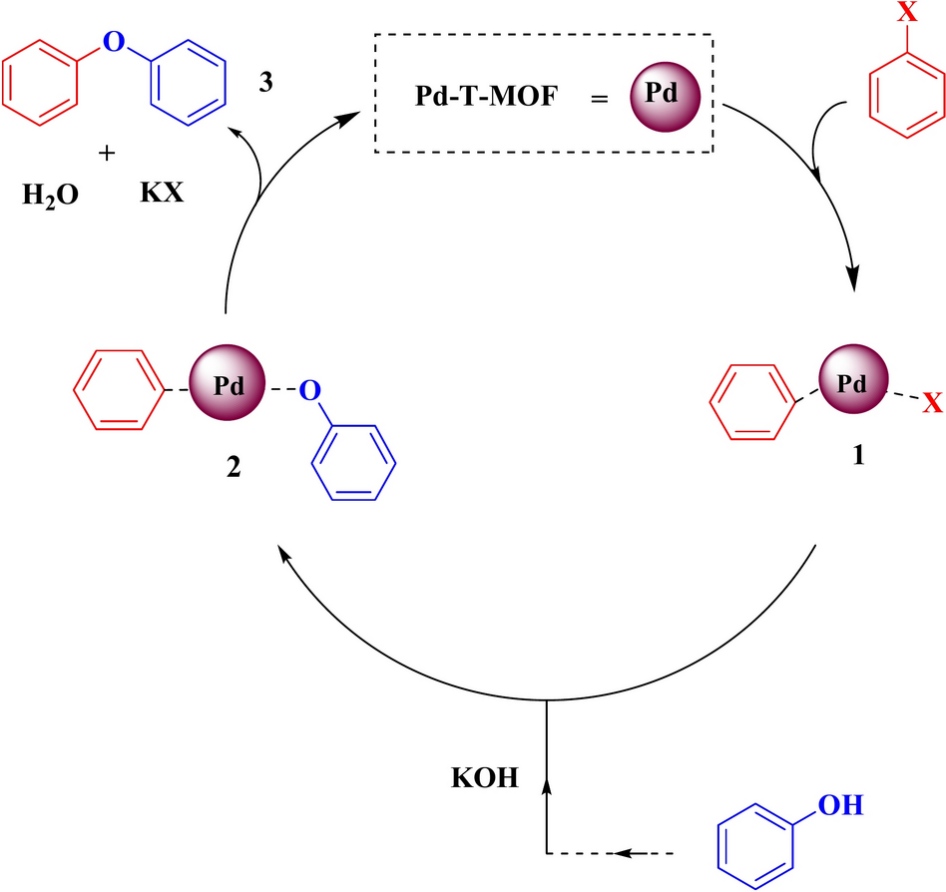
Proposed mechanism for C–O cross-coupling.

In Fig. 11, a cyclic mechanism is depicted to showcase the production of diaryl ethers using Pd-T-MOF NPs. The process encompasses oxidative addition, transmetallation, and reduction-elimination stages, resulting in the formation of diaryl ethers by reacting aryl halides with phenol. Additionally, the method includes catalyst regeneration to sustain the catalytic cycle.

### Hot filtration

To investigate the leaching of palladium in the reaction mixture and the heterogeneity of Pd-T-MOF, a hot filtration test was conducted during the synthesis of diaryl ethers through the coupling of iodobenzene and phenol. In this experiment, we achieved a product yield of 65% in half the time originally required for the reaction. The same reaction occurred again, and at the midpoint of the reaction, the catalyst was removed. The filtered solution was then left to react for 15 min. The yield of the reaction at this stage was 67%. Following the hot filtration, no additional reaction was observed, confirming the negligible leaching of palladium.

### Reusability of catalyst

**Fig. 12:**
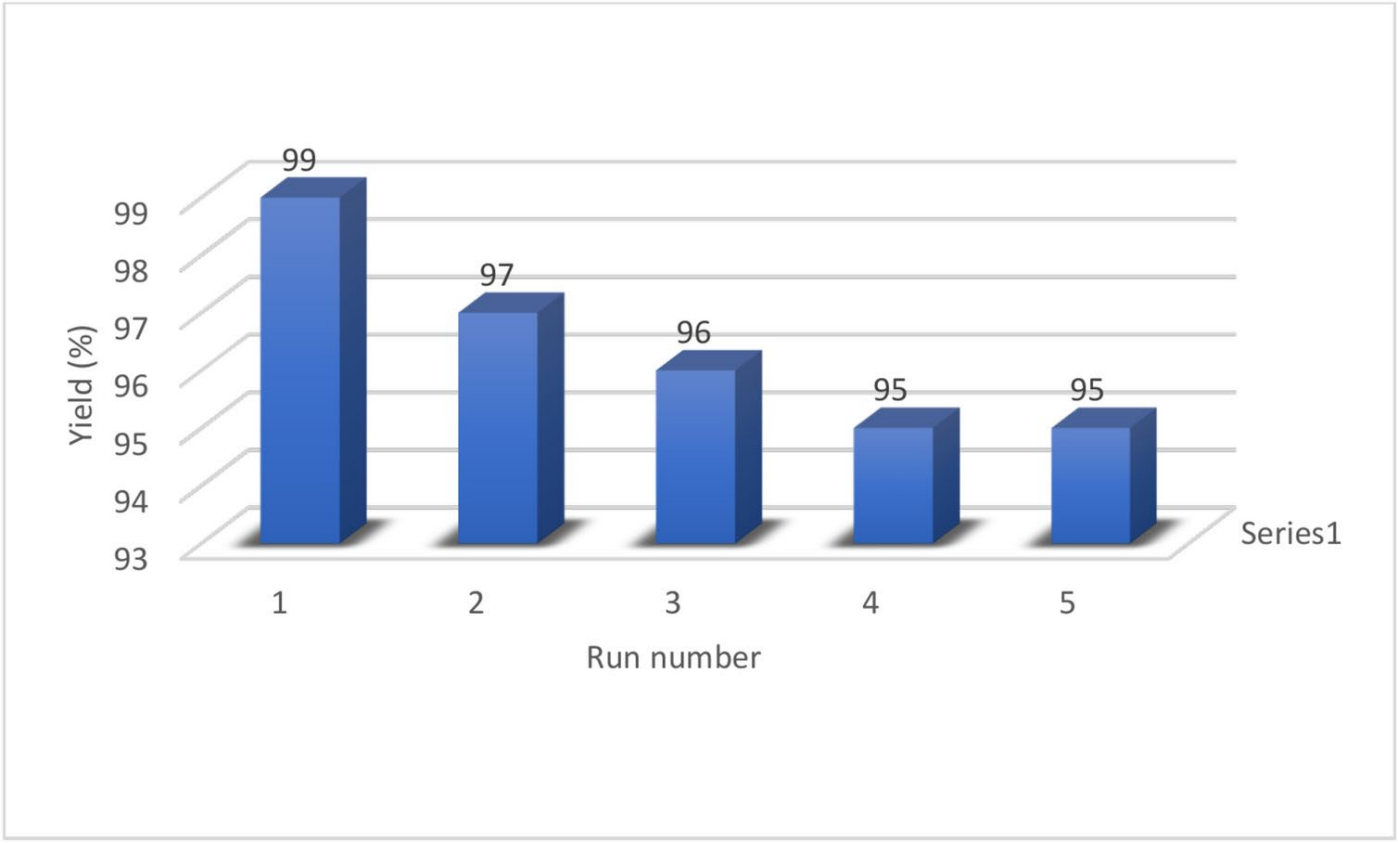
Recyclability of Pd-T-MOF.

The recyclability of catalysts is a crucial factor in catalytic systems. Thus, we examined the recyclability of Pd-T-MOF in the coupling reaction of phenol and iodobenzene (Fig. 12). Once the reaction was finished, the catalyst was separated using centrifugation, rinsed with ethyl acetate, and then reused up to 5 times without any significant decrease in its catalytic effectiveness. This clearly shows the practicality of recycling Pd-T-MOF.

Table 3 lists the activity and practicality of the Pd-T-MOF catalyst compared to other catalysts reported in the literature for the coupling of Ph-I with Ph-OH. As shown in Table 3, the synthesis of biphenyl ether resulted in higher yields when Pd-T-MOF was used as the catalyst compared to other catalysts. Thus, the Pd-T-MOF catalyst proves to be more practical, efficient, and capable of achieving higher isolated yields compared to alternative catalysts. Additionally, it allows for multiple recycling processes.

**Table 3:** Comparison of the catalytic efficiency of the prepared Pd-T-MOF in the C–O coupling reaction.

Entry	Catalyst	Reaction conditions	Time (min)	Yield (%)	Refs
1	Cu/RGO-Fe_3_O_4_ Cu nanoparticles	CsCO_3_, DMSO, 120 °C	12 h	98	^27^
2	AT-Nano CP Pd(0)	K_2_CO_3_, H_2_O, 60 °C	1 h	98	^28^
3	Household white light	Cs_2_CO_3_, DMSO, 80 °C	24 h	81	^29^
4	Fe_3_O_4_/CS-Cu	K_2_CO_3_, DMSO, 120 °C	15 h	95	^30^
5	CuO nanoparticles	KOH, DMSO, 110 °C, N_2_ atmosphere	15 h	93	^31^
6	Pd-T-MOF	KOH, EtOH, Reflux	30	98	This work

## Conclusions

In this study, a novel crystalline mesostructure of Pd-T-MOF was successfully synthesized using readily available starting materials, Pd(OAc)_2_ and trimesic acid. The resulting Pd-T-MOF nanocomposite demonstrated high yield and purity when employed as a nanocatalyst for C–O cross-coupling reactions. Comprehensive characterization of Pd-T-MOF was carried out using FT-IR, SEM, TGA, XRD, ICP, EDS, BET, and XPS techniques. The material proved to be an effective and recyclable heterogeneous catalyst for C–O coupling, showcasing impressive catalytic performance with yields reaching up to 98%. The catalytic activity was evaluated in ethanol (EtOH), chosen as a green solvent. Additionally, the protocol exhibited selectivity for aryl iodides and aryl bromides over aryl chlorides during the reaction. Key highlights of this method include its innovative approach, straightforward synthesis process, use of environmentally friendly solvents, stability, and reusability. Notably, the procedure also benefits from requiring only small amounts of catalyst, simplifying separation processes and allowing the nanocatalyst to be reused multiple times without significant loss of activity.

## Supplementary Information

Supplementary Information.

## Data Availability

All data generated or analyzed during this study are included in the published article and its supplementary information files.
