# Supplementary material for: A porous metal–organic framework (Pd-MOF) as an efficient and recyclable catalyst for the C–O cross-coupling reactions
Source: Sci Rep. 2025 Apr 16;15:13070. doi: 10.1038/s41598-025-97157-2 (PMC12003842; doi:10.1038/s41598-025-97157-2)
Supplement: Supplementary file 1 — Supplementary Information. [file 41598_2025_97157_MOESM1_ESM.docx]

**A porous metal-organic framework (Pd-MOF) as an efficient and recyclable catalyst for the C–O cross-coupling reactions**

**Figure S1. 1-chloro-4-phenoxybenzene** **(Table 2, entry 11)**:^1^H NMR (400 MHz, DMSO): δ_H_= 7.00- 7.29 (m, 10H) ppm.

**Figure S2.** **1-nitro-4-phenoxybenzene (Table 2, entry 5)**:^1^H NMR (400 MHz, DMSO): δ_H_= 7.44 (s, 3H), 7.10 (m, 6H) ppm.

**Figure S3.** **1-methoxy-3-phenoxybenzene (Table 2, entry 8)**:^1^H NMR (400 MHz, DMSO): δ_H_= 7.20 (d, 9H), 3.93 (s, 3H) ppm.

**Figure S4. 1-bromo-4-phenoxybenzene (Table 2, entry 3)**:^1^H NMR (400 MHz, DMSO): δ_H_= 7.0 (s, 4H), 6.7 (d, J= 12 Hz, 5H) ppm.

**Figure S5. Oxydibenzene (Table 2, entry 1)**:^1^H NMR (400 MHz, DMSO): δ_H_= 8.3-8.5 (m, 5H), 7.9 (m, 5H) ppm.

**Figure S6. 1-nitro-4-phenoxybenzene (Table 2, entry 9)**:^1^H NMR (400 MHz, DMSO): δ_H_= 8.1 (m, 6H), 7.9 (m, 3H) ppm.

**Figure S7. Oxydibenzene (Table 2, entry 6)**:^1^H NMR (400 MHz, DMSO): δ_H_= 8.0 (m, 5H), 7.4 (m, 5H) ppm.

**Figure S8. 4-phenoxyphenol (Table 2, entry 4)**:^1^H NMR (400 MHz, DMSO): δ_H_= 7.1-7.4 (m, 9H), 4.7 (s, 1H) ppm.

**Figure S9. 1-methyl-4-phenoxybenzene (Table 2, entry 2)**:^1^H NMR (400 MHz, DMSO): δ_H_= 7.0-7.2 (m, 9H), 2.1 (s, 3H) ppm.

**Figure S10. 1-nitro-4-phenoxybenzene (Table 2, entry 13)**:^1^H NMR (400 MHz, DMSO): δ_H_= 7.6 (m, 4H), 7.2 (s, 5H) ppm.

**Figure S11. 1-methoxy-4-phenoxybenzene (Table 2, entry 7)**:^1^H NMR (400 MHz, DMSO): δ_H_= 7.1 (m, 5H), 6.9 (m, 4H), 4.0 (s, 3H) ppm.


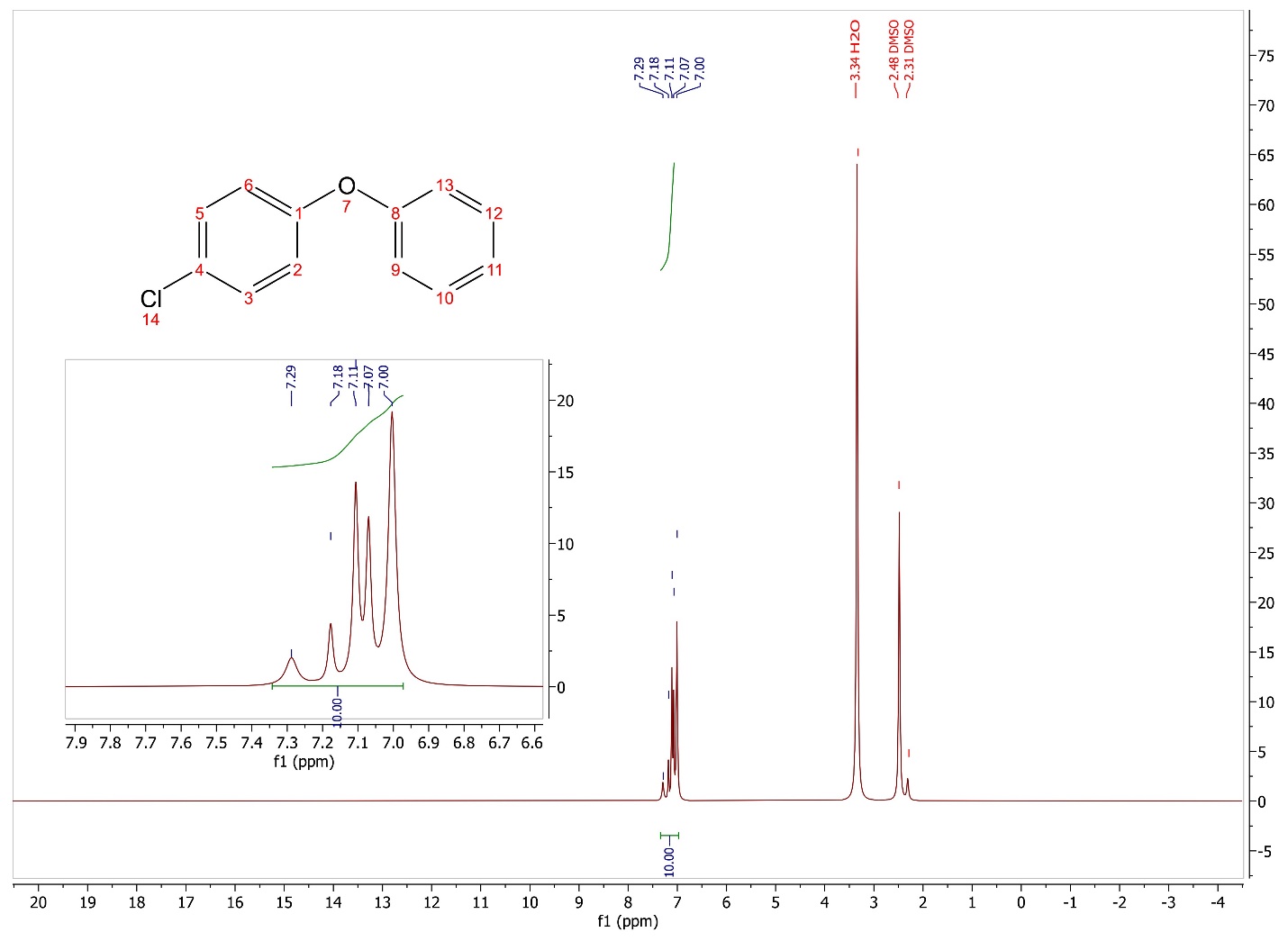


**Figure S1. 1-chloro-4-phenoxybenzene (Table 2, entry 11)**


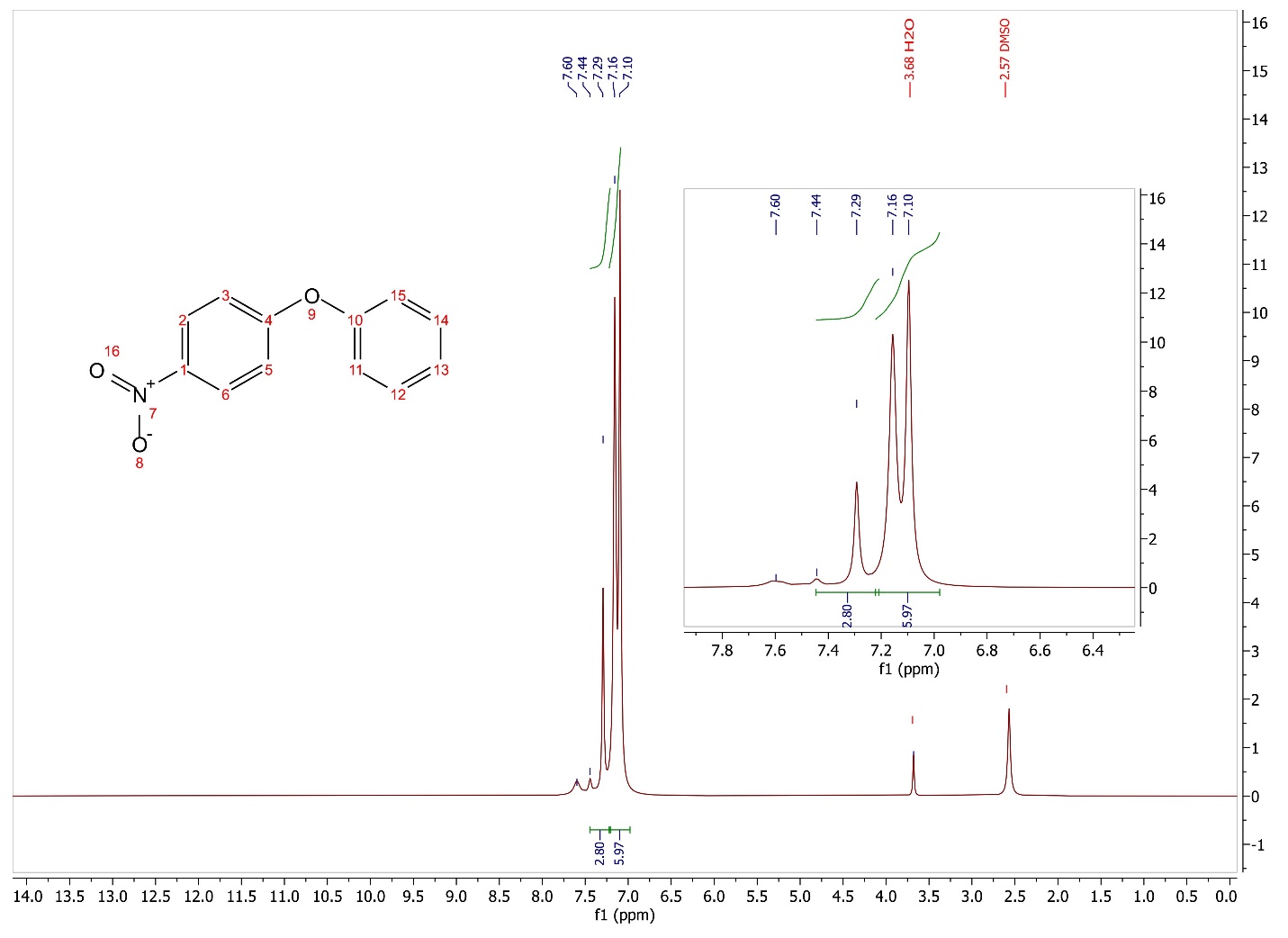


**Figure S2. 1-nitro-4-phenoxybenzene (Table 2, entry 5)**

**
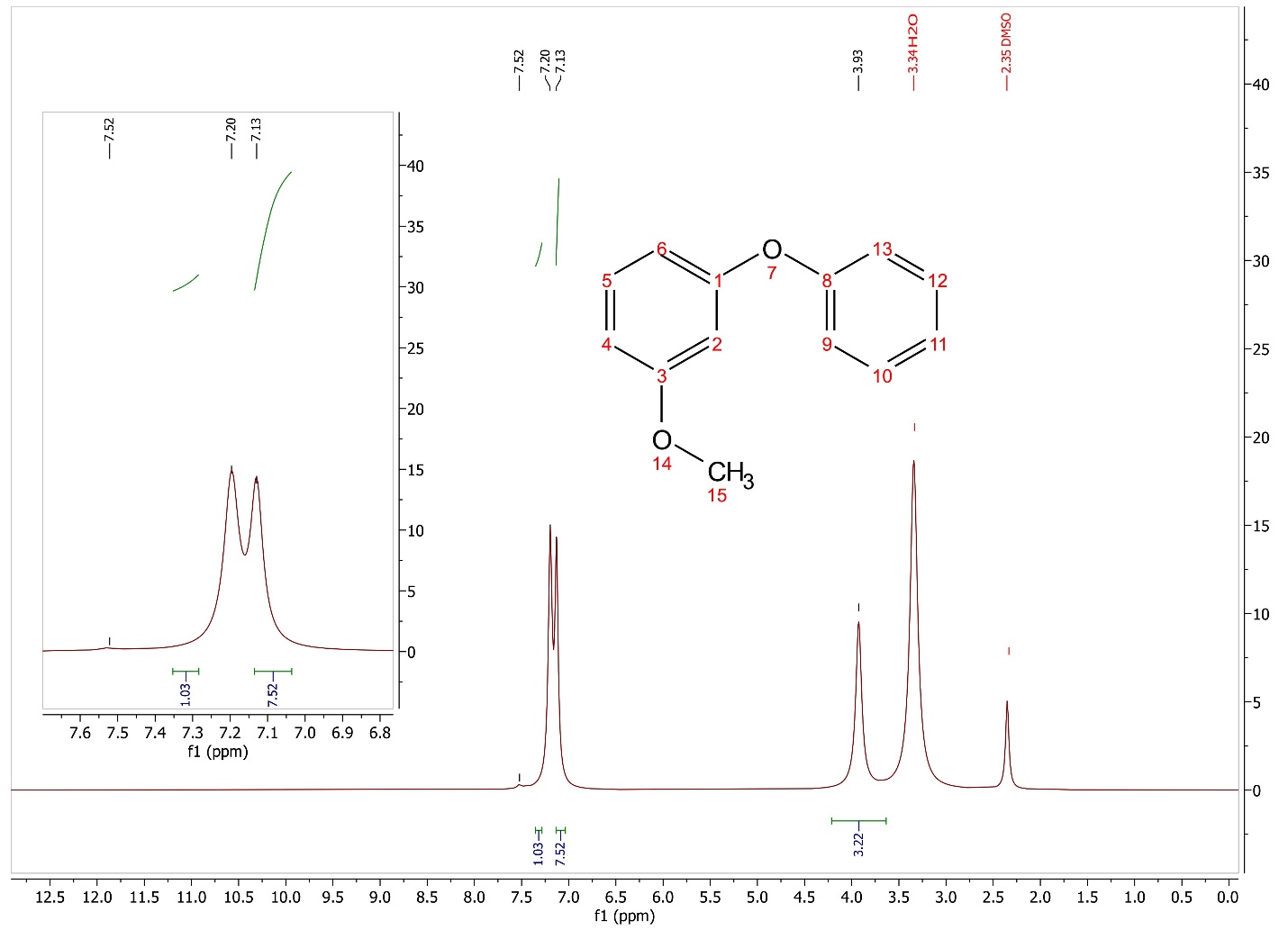
**

**Figure S3. 1-methoxy-3-phenoxybenzene (Table 2, entry 8)**

**
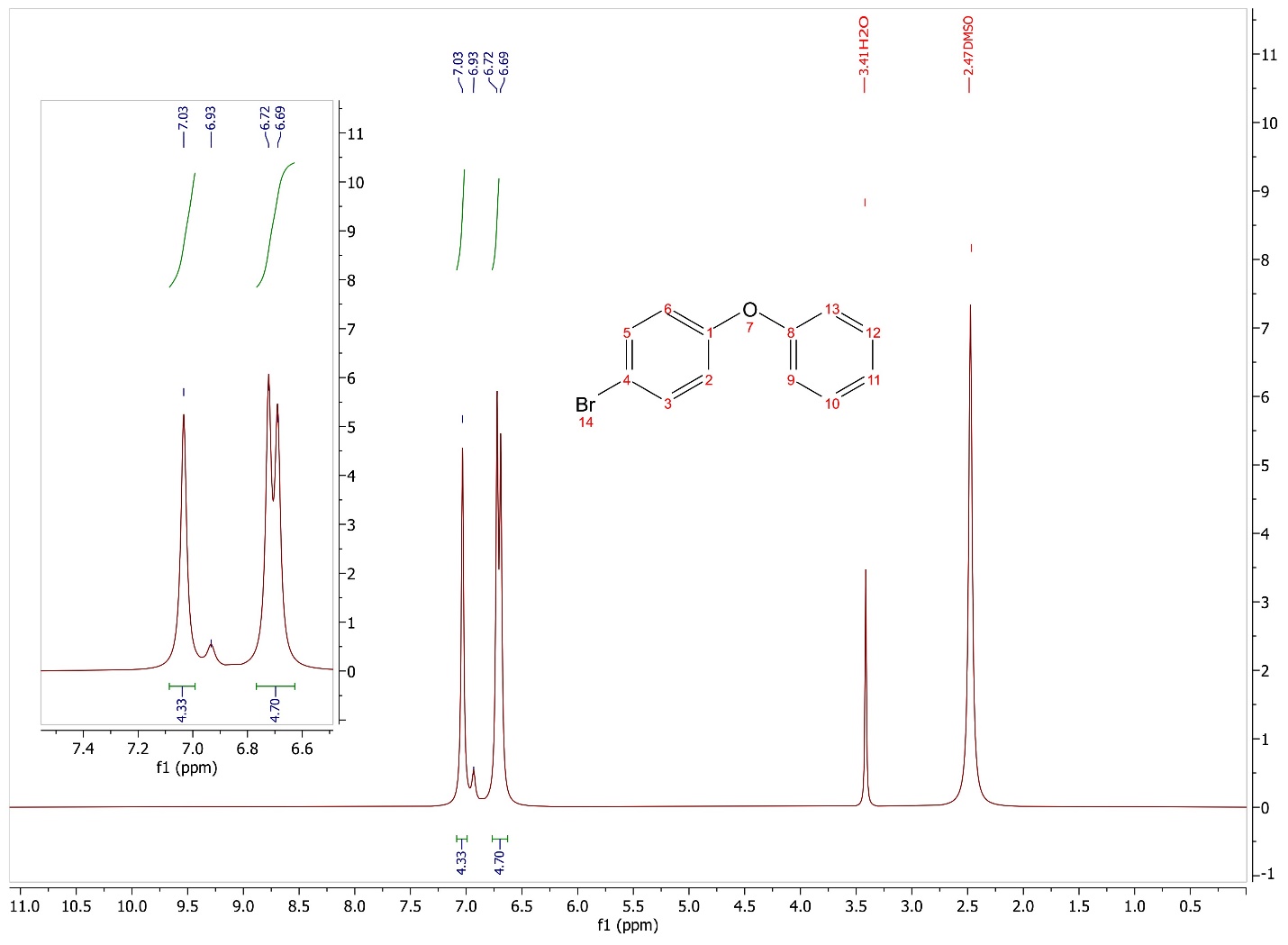
**

**Figure S4. 1-bromo-4-phenoxybenzene (Table 2, entry 3)**


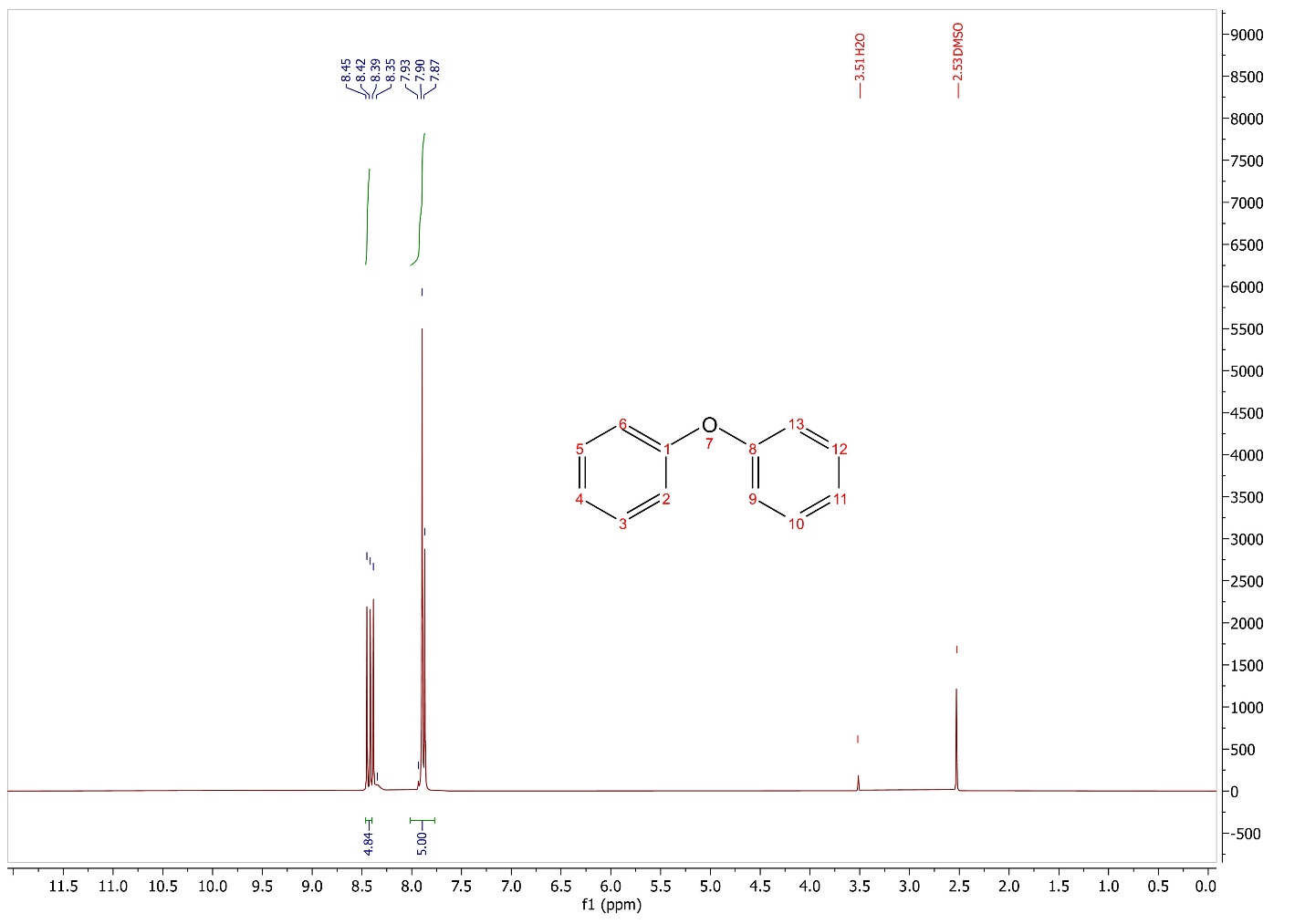


**Figure S5. Oxydibenzene (Table 2, entry 1)**

**^
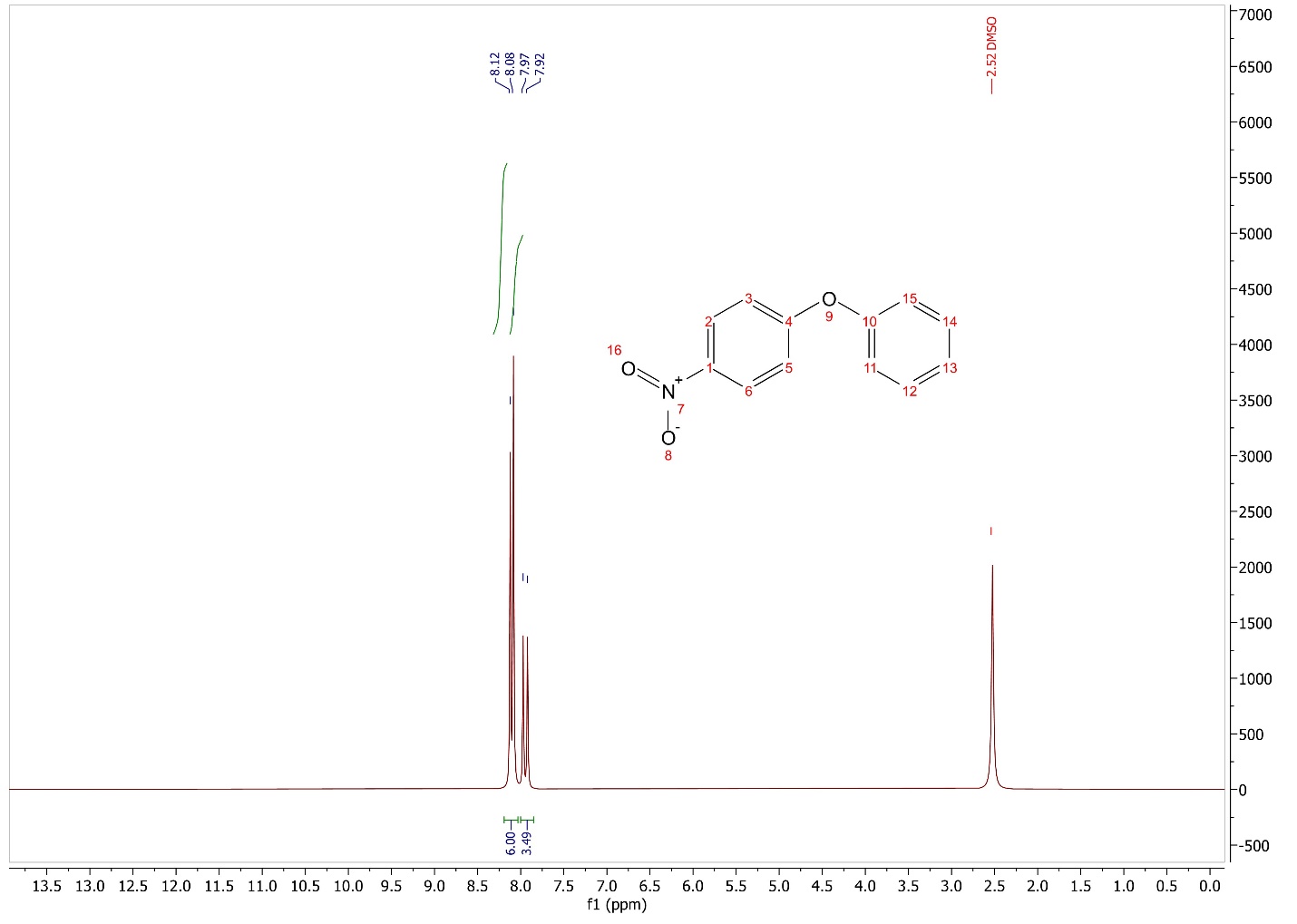
^**

**Figure S6. 1-nitro-4-phenoxybenzene (Table 2, entry 9)**

**
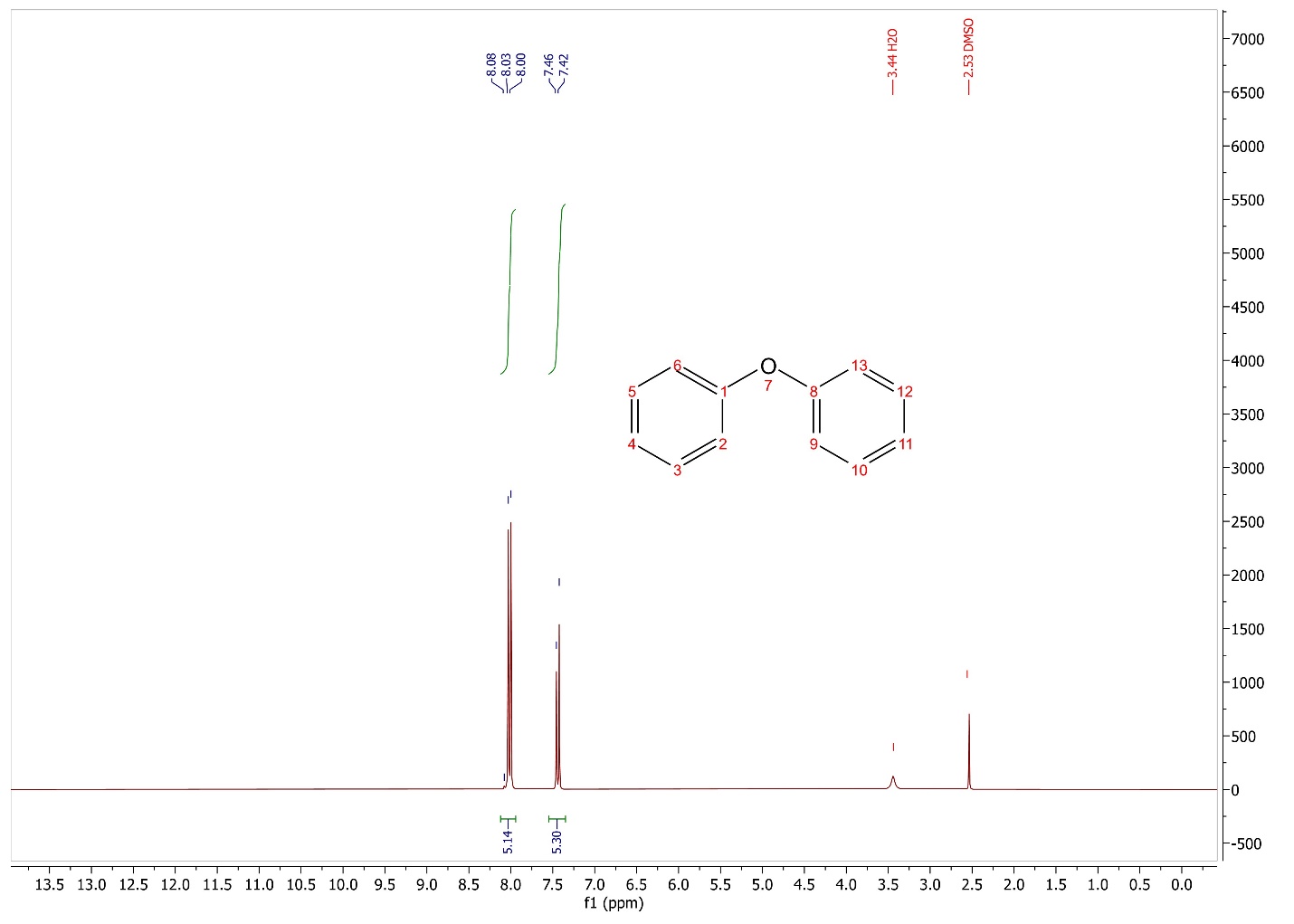
**

**Figure S7. Oxydibenzene (Table 2, entry 6)**

**
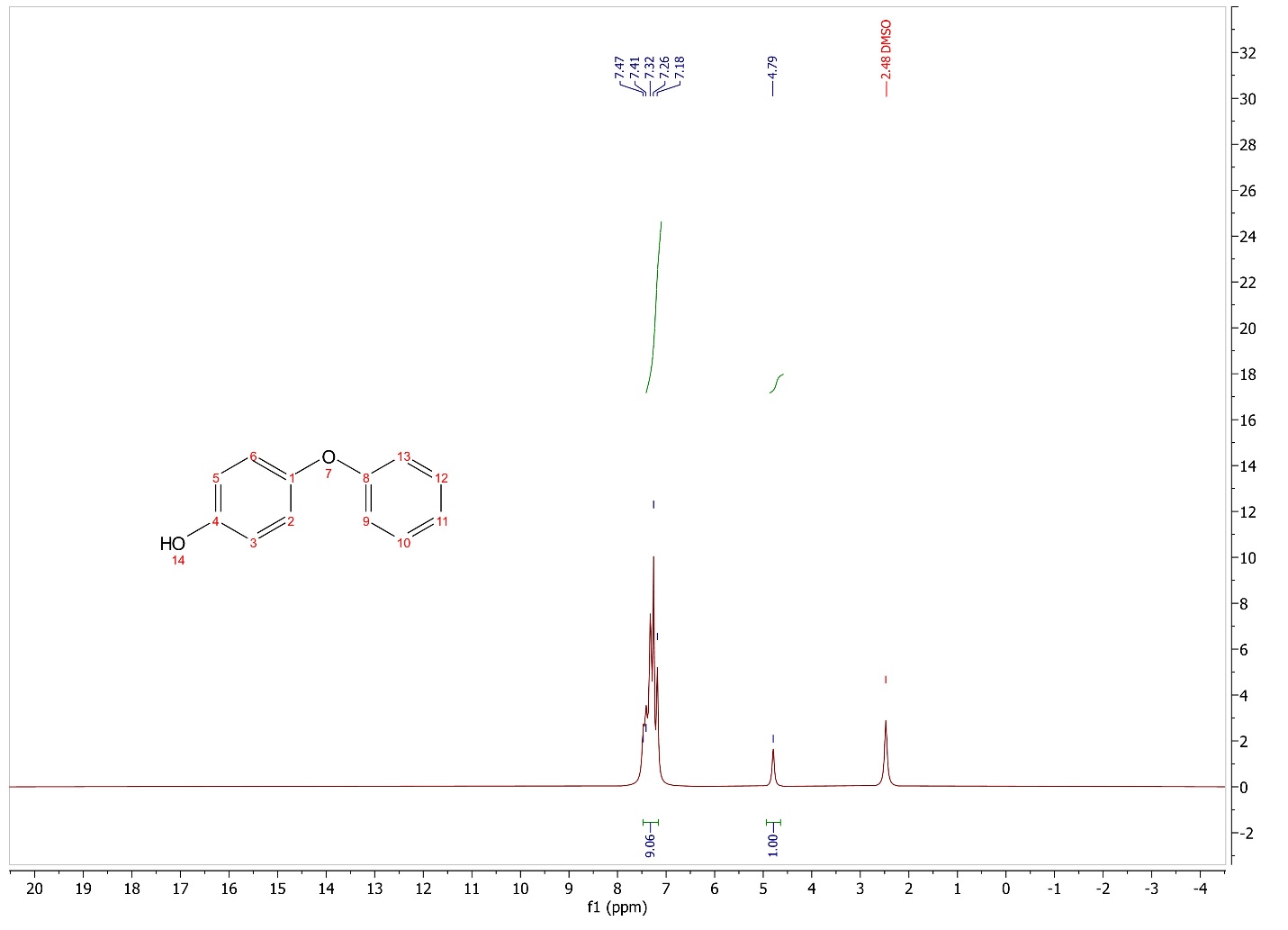
**

**Figure S8. 4-phenoxyphenol (Table 2, entry 4)**


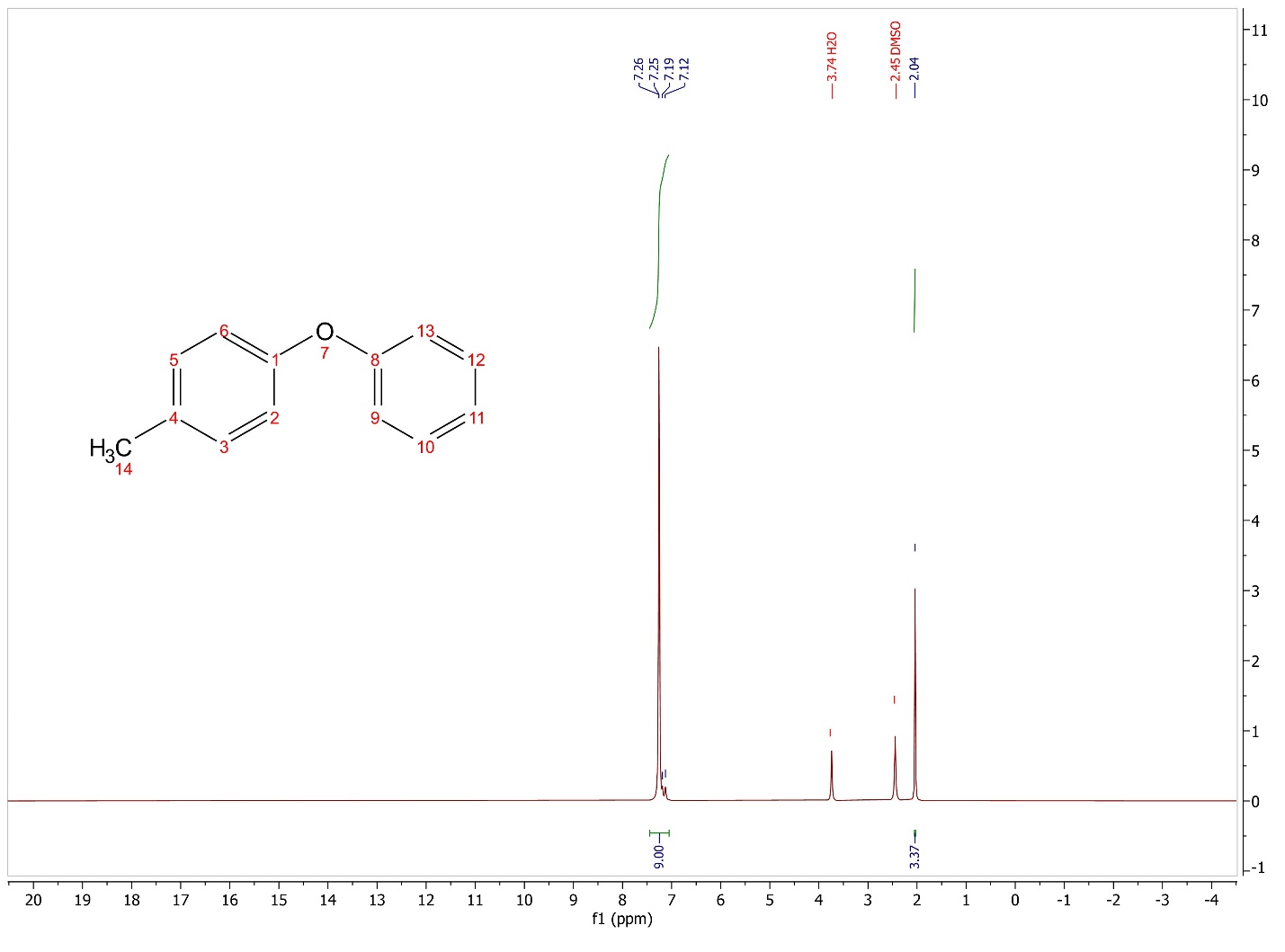


**Figure S9. 1-methyl-4-phenoxybenzene (Table 2, entry 2)**

**
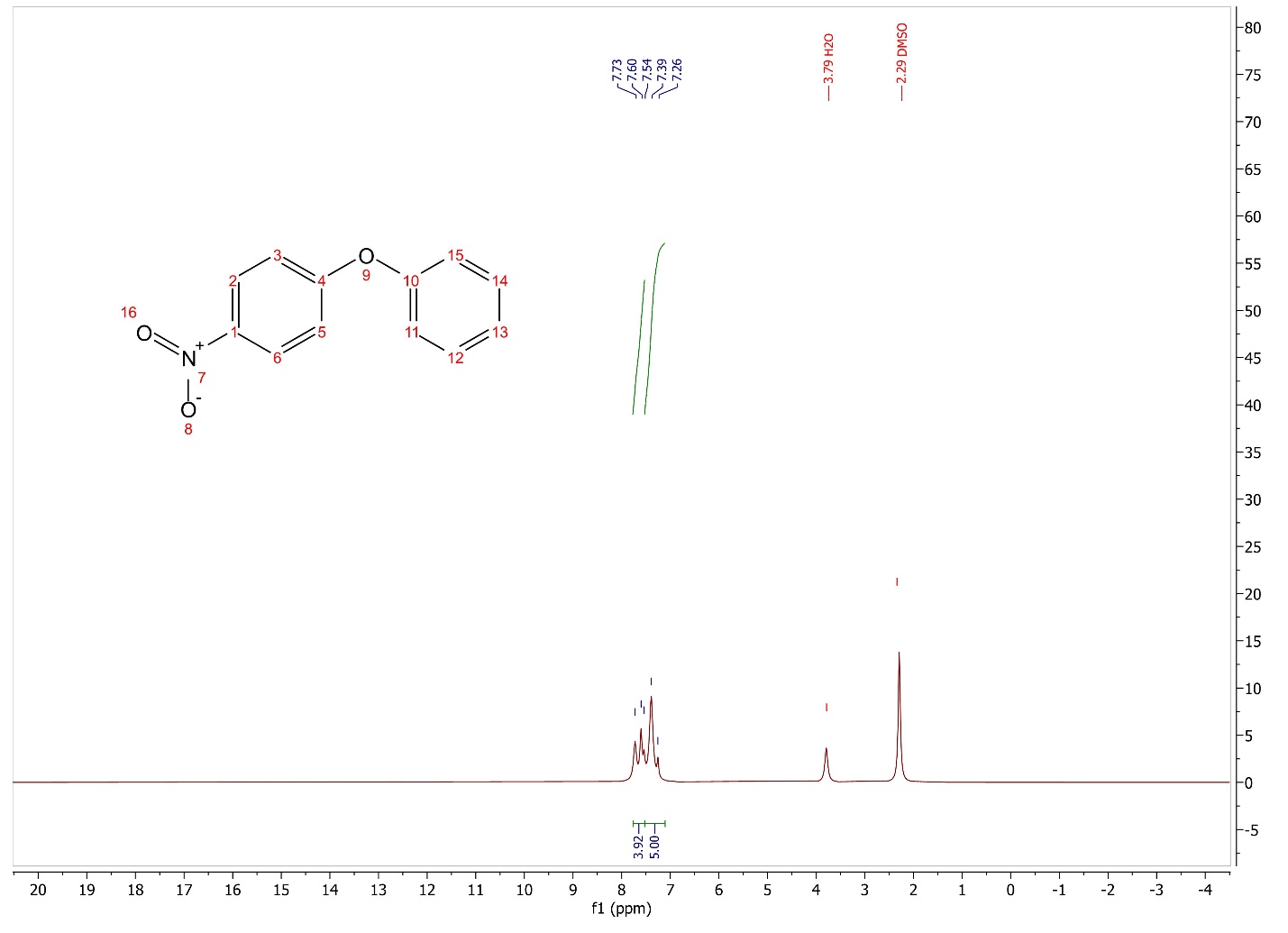
**

**Figure S10. 1-nitro-4-phenoxybenzene (Table 2, entry 13)**


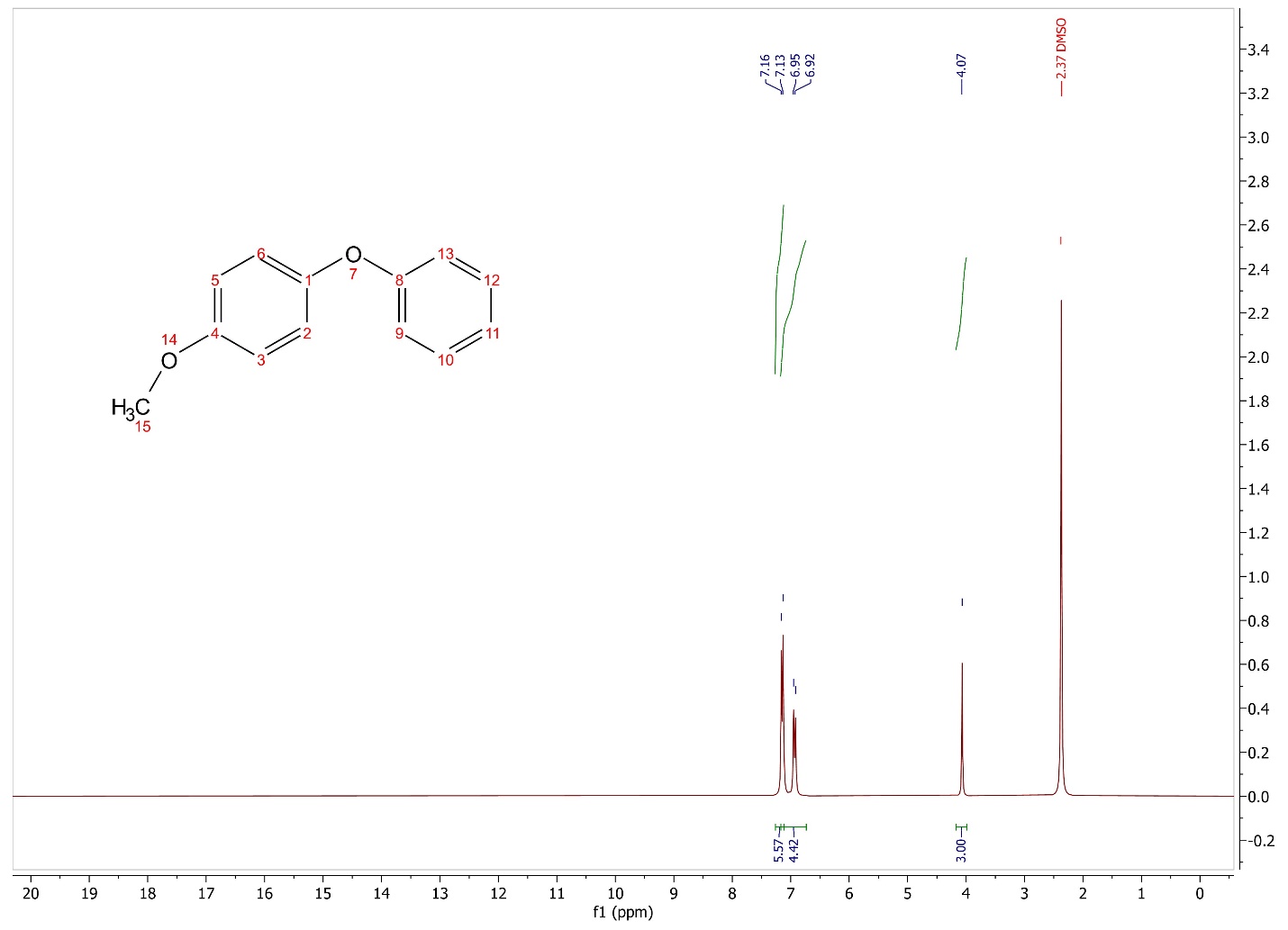


**Figure S11. 1-methoxy-4-phenoxybenzene (Table 2, entry 7)**
